# Supplementary material for: The lower jaw of Devonian ray‐finned fishes (Actinopterygii): Anatomy, relationships, and functional morphology
Source: Anat Rec (Hoboken). 2025 Jul 21;309(3):550–602. doi: 10.1002/ar.70005 (PMC12882060; doi:10.1002/ar.70005)
Supplement: Supplementary file 1 — Supplementary Figure 1. Mandibles of Cheirolepis trailli NHMUK PV P 1370 prior to retrodeformation. (a) Right mandible in medial view. (b) Right mandible in dorsal view. Scale bar = 5 mm. Supplementary Figure 2. Mandibles of Cheirolepis jonesi, PMO 235.121 prior to retrodeformation. (a) Left mandible in lateral view. (b) Right mandible in lateral view. Scale bar = 5 mm. Supplementary Figure 3. Mandibles of Limnomis delaneyi ANSP 23721 prior to retrodeformation. (a) Left mandible in lateral view. (b) Right mandible in lateral view. Scale bar = 1 mm. Table S1: Overview of Devonian actinopterygians. Table S2: Taxa excluded from the study due to unsuitability of CT scanning. Table S3: Taxa included in study and CT scanning parameters. Table S4: Data availability. Temporary Dropbox links are provided for the PLY files during the review process, and will be replaced with stable Morphosource DOIs upon acceptance. [file AR-309-550-s001.docx]

**Table 1: Overview of Devonian actinopterygians**

| **Name** | **Previous literature** | **Included in study** | **Reason for exclusion** | **Stage** | **Locality** | **Stratigraphy** |
| --- | --- | --- | --- | --- | --- | --- |
| *Meemannia eos* | Zhu et al., 2006, 2010; Lu et al., 2016 | Yes | n/a | Lochkovian | Xitun, Qujing, East Yunnan, China | Xitun Formation, Qujing |
| *Cheirolepis trailli* | Pearson & Westoll, 1979; Giles et al., 2015a | Yes | n/a | Eifelian | Tynet Burn; Clune; Lethen Burn; Orkey, Scotland | Achanarras Fish Bed Member, Lybster Flagstone Formation |
| *?Howqualepis youngorum* | Choo, 2009 | No | Lower jaw not preserved | Eifelian | New South Wales, Australia | Bunga Beds |
| *Austelliscus ferox* | Figueroa et al., 2021 | Yes | n/a | Eifelian- Givetian | Parana, Brazil | Ponta Grossa Formation |
| *Donnrosenia schaefferi* | Long et al., 2008 | No | Not amenable to CT scanning (size, density) | Givetian | Southern Victoria Land, Antarctica | Aztec Siltstone |
| *Howqualepis rostridens* | Long, 1998 | Yes | n/a | Givetian | Upper Howqua River, Mount Howitt, Australia | Lower mudstone unit, Avon River Supergroup |
| *Cheirolepis jonesi* | Newman et al., 2021 | Yes | n/a | Givetian | Estheriahaugen North, Spitsbergen, Svalbard | Fiskeløfta Member, Tordalen Formation, Mimerdalen Subgroup |
| *Cheirolepis schultzei* | Arratia & Cloutier, 2004 | No | Lower jaw incomplete | Givetian | Red Hill, Horse Creek Valley, Eureka County, central Nevada, USA | Red Hills Beds |
| *Stegotrachelus finlayi* | Gardiner, 1963; Swartz, 2009 | No | Not amenable to CT scanning (size) | Givetian | Ness of Sound & Dunrossness, Shetland, Scotland | Brindister Flags Formation, Middle Old Red Sandstone |
| *Moythomasia lineata* | Jessen, 1968; Choo, 2015 | No | Not amenable to CT scanning (size, density) | Frasnian | Heiligenstock Quarry, North Rhine-Westphalia, Germany | Oberer Plattenkalk Member, Ahrdorf Formation |
| *Moythomasia nitida* | Jessen, 1968; Choo ,2015 | No | Not amenable to CT scanning (flattened, density) | Givetian- Frasnian | Heiligenstock Quarry, North Rhine-Westphalia, Germany | Oberer Plattenkalk Member, Ahrdorf Formation |
| *Mimipiscis toombsi* | Gardiner, 1984;  Choo, 2012 | Yes | n/a | Frasnian | Paddy’s Valley, Mt. Pierre Station, Western Australia | Gogo Formation |
| *Mimipiscis bartrami* | Gardiner, 1984;  Choo, 2012 | Yes | n/a | Frasnian | Paddy’s Valley, Mt. Pierre Station, Western Australia | Gogo Formation |
| *Moythomasia durgaringa* | Gardiner, 1984;  Choo, 2015 | Yes | n/a | Frasnian | Paddy’s Valley, Mt. Pierre Station, Western Australia | Gogo Formation |
| *Gogosardina coatesi* | Choo et al., 2009a | Yes | n/a | Frasnian | Paddy’s Valley, Mt. Pierre Station, Western Australia | Gogo Formation |
| *Pickeringius acanthophorus* | Choo et al., 2019 | No | Lower jaw incomplete | Frasnian | Paddy’s Valley, Mt. Pierre Station, Western Australia | Gogo Formation |
| *Moythomasia perforata* | Gross, 1942 | No | Lower jaw not preserved | Frasnian | Kokenhusen (Koknese), Latvia | Snetnaya Group |
| *Cheirolepis canadensis* | Arratia & Cloutier, 1996; Arratia, 2009. | No | Not amenable to CT scanning (size, preservation) | Frasnian | River Ristigouche, Quebec, Canada | Escuminac Formation |
| *Raynerius splendens* | Giles et al., 2015b | Yes | n/a | Frasnian | La Parisienne quarry, Pas-de-Calais, France | Grey Member, Ferques Formation |
| “*Moythomasia*” *devonica* | Clarke, 1885; Hussakof & Bryant, 1918; Gardiner, 1963 | Yes | n/a | Frasnian | Folsomdale, New York State, USA | Rhinestreet Shale, West Falls Formation |
| *Krasnoyarichthys jesseni* | Prokofiev, 2002 | No | Lower jaw not preserved | Famennian | Preobrazhensky Village, Nazarovo City, Krasnoyarski Krai, Russia | Unknown |
| *Osorioichthys marginis* | Taverne, 1997 | Yes | n/a | Famennian | Philippeville-Mariembourg Le Fraity Highway, Belguim | Famenne Shales |
| *Palaeoneiros clackorum* | Eastman, 1907; Giles et al., 2022 | Yes | n/a | Famennian | Warren, Pennsylvania, USA | Chadakoin Formation |
| “*Kentuckia*” *hlavini* | Dunkle, 1964; Feldman, 1996 | Yes | n/a | Famennian | Cuyahoga County, Ohio, USA | Cleveland Shale Member, Ohio Shale |
| Actinopterygii n gen n sp | Friedman & Blom, 2006 | Yes | n/a | Famennian | Cuyahoga County, Ohio, USA | Cleveland Shale Member, Ohio Shale |
| *Tegeolepis clarki* | Gardiner, 1963; Dunkle & Schaeffer, 1973;  Figueroa et al., 2021 | Yes | n/a | Famennian | Cleveland, Ohio, USA | Cleveland Shale Member, Ohio Shale |
| *Limnomis delaneyi* | Daeschler, 2000 | Yes | n/a | Famennian | Red Hill site, nr Hyner, Clinton County, Pennsylvania, USA | Duncannon Member, Catskill Formation |
| “*Gonatodus*” *brainerdi* | Newberry, 1889 | Yes | n/a | Famennian | Chagrin Falls, Ohio, USA | ?Berea Sandstone, Appalachian Basin |
| *Cuneognathus gardineri* | Friedman & Blom, 2006 | Yes | n/a | Latest Famennian/earliest Tournaisian | Celsius Bjerg, Ymer ÿ, East Greenland | Obrutschew Bjerg Formation |

**Table 2: Taxa excluded from the study due to unsuitability of CT scanning**

| **Name** | **Specimen no.** | **Specimen info** | **Voltage (kV)** | **Current (uA)** | **Exposure (ms)** | **projections** | **frames per projection** | **filter** | **voxel size** | **Minimise ring artefacts** |
| --- | --- | --- | --- | --- | --- | --- | --- | --- | --- | --- |
| *Donnrosenia schaefferi* | NHMUK PV P 12591 | Isolated left mandible from Granite Harbour, Antarctica; indet ‘palaeoniscid’, almost certainly attributable to *D. schaefferi* | 154 | 78 | 1415 | 3442 | 4 | 0.5mm Cu | 12.61 μm | No |
|  | Tomogram stack (.TIFF):  <https://doi.org/10.17602/M2/M683257> | | | | | | | | | |
| *Moythomasia lineata* | NRM P.6191 | Disarticulated cranium | 160 | 62 | ? | ? | ? | ? | 52.35 μm | ? |
|  | Tomogram stack (.TIFF):  <https://doi.org/10.17602/M2/M685370> | | | | | | | | | |
| *Moythomasia nitida* | NHMUK PV P 53578 | Articulated cranium | 185 | 65 | 500 | 3274 | 2 | 1.0mm Cu | 10.00 μm | No |
|  | Tomogram stack (.TIFF):  <https://doi.org/10.17602/M2/M683421> | | | | | | | | | |
| *Cheirolepis canadensis* | MCZ VPF 6709 | Partially articulated cranium | 110 | 300 | 485 | 1528 | 1 | 0.3mm Cu | 34.09 μm | Yes |
|  | Tomogram stack (.TIFF):  <https://doi.org/10.17602/M2/M689375> | | | | | | | | | |

**Table 3: Taxa included in study and CT scanning parameters**

| **Name** | **Specimen no.** | **Specimen info** | **Mandible** | **Voltage (kV)** | **Current (uA)** | **Exposure (ms)** | **projec-tions** | **frames per proj** | **filter** | **voxel size** | **Min. ring artefacts** |
| --- | --- | --- | --- | --- | --- | --- | --- | --- | --- | --- | --- |
| *Meemannia eos* | IVPP V14536.5 | Isolated mandible | Left | 110 | 120 | 1000 | 1440 | 2 | 1.00 mm Al | 8.62 μm | No |
| *Cheirolepis trailli* | NHMUK  PV P 62908b | Semi-articulated specimen | Right | See materials and methods (synchrotron tomography). | | | | | | | |
|  | NHMUK PV P 1370 | Semi-articulated specimen | Right (and left articular) | 215 | 130 | 1000 | 4476 | 8 | 1.0 mm Sn | 27.33 μm | No |
| *Austelliscus ferox* | MCT890-P | Isolated mandible (mouldic) | Left | Scan data from Figueroa et al. (2021). | | | | | | | |
| *Howqualepis rostridens* | AMF65495 | Semi-articulated specimen (mouldic) | Right | Scan data from Giles et al. (2015a). | | | | | | | |
|  | MV P.160801 | Poorly-articulated specimen; mouldic | Left (and right anterior partial) | 175 | 314 | 2000 | 2300 | 1 | 0.5 mm Sn | 55 μm | No |
| *Cheirolepis jonesi* | PMO 235.121 | Semi-articulated specimen | Both | Scan data from Newman et al. (2021). | | | | | | | |
| *Mimipiscis toombsi* | NHMUK PV P 56495 | Isolated mandible (due to acid prep.) | Right | 110 | 50 | 1000 | 4476 | 1 | 0.1 mm Cu | 6.26 μm | No |
|  | NHMUK PV P 53249 | Acid prepared cranium | Right | 80 | 238 | 500 | 4476 | 1 | 0.1 mm Cu | 20.26 μm | No |
| *Mimipiscis bartrami* | NHMUK PV P 53254 | Isolated mandible (due to acid prep.) | Right | 79 | 228 | 500 | 4476 | 1 | 0.1 mm Cu | 19.46 μm | no |
| *Moythomasia durgaringa* | AMNH FF 11598 | Articulated specimen (acid prepared) | Right | 150 | 165 | 708 | 3141 | 2 | None | 27.68 μm | no |
| *Gogosardina coatesi* | MV P.228269 | Isolated mandible (due to acid prep.) | Left | 150 | 107 | 1000 | 700 | 2 | 0.5 mm Al | 16 μm | No |
| *Raynerius splendens* | MGL 1245 | Articulated specimen | Both | Scan data from Giles et al. (2015b). | | | | | | | |
| “*Moythomasia*” *devonica* | BSNS E22113 | Isolated mandible | Left | 155 | 105 | 1415 | 3141 | 2 | None | 18.13 μm | No |
| *Osorioichthys marginis* | IRSNB P 1340 | Articulated specimen | Right |  |  |  |  |  |  |  |  |
| *Palaeoneiros clackorum* | MCZ VPF-5114 | Articulated specimen | Both | Scan data from Giles et al. (2023). | | | | | | | |
| “*Kentuckia*” *hlavini* | CMNH 9562 | Isolated mandible | Left | 145 | 130 | 1000 | 3141 | 1 | 0.1 mm Cu | 23.71 μm | no |
| Actinopterygii n. gen. n. sp. | CMNH 9560 | Isolated mandible | Right | 135 | 125 | 1000 | 3141 | 1 | 0.1 mm Cu | 17.86 μ | No |
| *Tegeolepis clarki* | CMNH 8124 | Isolated mandible and fragments | Right | Scan data from Figueroa et al. (2021). | | | | | | | |
|  | NHMUK PV P 45312 | Semi-articulated specimen | Right | 205 | 225 | 1415 | 4150 | 2 | 2.0 mm Cu | 122.1 μm | Yes |
| *Limnomis delaneyi* | ANSP 23721 | Semi-articulated specimen | Both | 80 | 80 | 4000 | 3141 | 2 | 1.4 mm Al | 6.53 μm | Yes |
| “*Gonatodus*” *brainerdi* | ANSP 6232 | Articulated specimen | Left | 155 | 155 | 2829 | 3141 | 4 | 1.25mm Cu | 24.03 μm | Yes |
| *Cuneognathus gardineri* | NHMD-1235389 | Semi-articulated specimen | Both | 105 | 110 | 2829 | 3141 | 2 | 0.25 mm Cu | 12.01 μm | Yes |

**Table 4: Data availability**

Temporary Dropbox links are provided for the PLY files during the review process, and will be replaced with stable Morphosource DOIs upon acceptance.

| **Taxon** | **Media type and DOI** |
| --- | --- |
| *Meemannia eos* IVPP V14536.5 | Tomogram stack (.TIFF) and 3D surface file left mandible (.PLY):  [http://admorph.ivpp.ac.cn/client/fossil/details/Fish/4771afe5324141e7b67ebc1e0e8b876a](https://wx.qq.com/cgi-bin/mmwebwx-bin/webwxcheckurl?requrl=http%3A%2F%2Fadmorph.ivpp.ac.cn%2Fclient%2Ffossil%2Fdetails%2FFish%2F4771afe5324141e7b67ebc1e0e8b876a&skey=%40crypt_fa799926_b4a1d16039152fdf42209d8b6fad240e&deviceid=e741958234005675&pass_ticket=xMhLmU3DY%252FV1RLKGtR9LopKDRIhRU3nGFPUYjLDYhVvc0Rwi5Rt30zm3bM2y1kt5DnWQjalGZBjPwIRlDcMDmA%253D%253D&opcode=2&scene=1&username=@9c8a5e75b81ed54807a89b378c7a1dcc) |
| *Cheirolepis trailli* NHMUK  PV P 62908b | Projections (.TIFF):  <https://doi.org/10.15151/ESRF-DC-2013017890>  (.TIFF):  <https://doi.org/10.17602/M2/M699694>  3D surface file right mandible (.PLY):  https://doi.org/[10.17602/M2/M699729](https://doi.org/10.17602/M2/M699729) |
|  |  |
| *Cheirolepis trailli* NHMUK PV P 1370 | Tomogram stack (.TIFF):  <https://doi.org/10.17602/M2/M666828>  3D surface file right mandible (.PLY):  <https://doi.org/>[10.17602/M2/M666948](https://doi.org/10.17602/M2/M666948)  3D surface file left articular (.PLY):  https://doi.org/[10.17602/M2/M673174](https://doi.org/10.17602/M2/M673174) |
| *Austelliscus ferox* MCT890-P | Scan data from Figueroa et al. (2021):  <https://doi.org/10.5061/dryad.b8gtht7b7> |
| *Howqualepis rostridens* AMF65495 | Tomogram stack (.TIFF):  <https://doi.org/10.17602/M2/M669275>  3D surface file right mandible (.PLY):  https://doi.org/1[0.17602/M2/M669280](https://doi.org/10.17602/M2/M669280) |
|  |  |
| *Howqualepis rostridens* MV P.160801 | Tomogram stack (.TIFF):  <https://doi.org/10.17602/M2/M668627>  3D surface file left mandible (.PLY):  https://doi.org/[10.17602/M2/M671466](https://doi.org/10.17602/M2/M671466)  3D surface file right mandible (.PLY):  https://doi.org/[10.17602/M2/M671463](https://doi.org/10.17602/M2/M671463) |
| *Cheirolepis jonesi* PMO 235.121 | Scan data from Newman et al. (2021):  <https://dx.doi.org/10.5281/zenodo.4431685>  3D surface file reconstructed left mandible (.PLY):  https://doi.org/ [10.17602/M2/M699748](https://doi.org/10.17602/M2/M699748)  3D surface file reconstructed right mandible (.PLY):  https://doi.org/[10.17602/M2/M699752](https://doi.org/10.17602/M2/M699752) |
| *Mimipiscis toombsi* NHMUK PV P 56495 | Projections (.TIFF):  <https://doi.org/10.17602/M2/M668350>  Tomogram stack (.TIFF):  <https://doi.org/10.17602/M2/M668365>  3D surface file right mandible (.PLY):  https://doi.org/[10.17602/M2/M668384](https://doi.org/10.17602/M2/M668384) |
|  |  |
| *Mimipiscis toombsi* NHMUK PV P 53249 | Projections (.TIFF):  <https://doi.org/10.17602/M2/M668541>  Tomogram stack (.TIFF):  <https://doi.org/10.17602/M2/M668591>  3D surface file right mandible (.PLY):  https://doi.org/[10.17602/M2/M668620](https://doi.org/10.17602/M2/M668620) |
| *Mimipiscis bartrami* NHMUK PV P 53254 | Tomogram stack (.TIFF):  <https://doi.org/10.17602/M2/M669117>  3D surface file right mandible (.PLY):  https://doi.org/[10.17602/M2/M669120](https://doi.org/10.17602/M2/M669120) |
| *Moythomasia durgaringa* AMNH FF 11598 | Tomogram stack (.TIFF):  <https://doi.org/10.17602/M2/M566968>  3D surface file right mandible (.PLY):  <https://www.morphosource.org/concern/media/000671760?locale=en> |
| *Gogosardina coatesi* MV P.228269 | Tomogram stack (.TIFF):  <https://doi.org/10.17602/M2/M670508>  3D surface file left mandible (.PLY):  https://doi.org/[10.17602/M2/M670517](https://doi.org/10.17602/M2/M670517)  3D surface file left mandible (.OBJ):  https://doi.org/[10.17602/M2/M673184](https://doi.org/10.17602/M2/M673184) |
| *Raynerius splendens* MGL 1245 | Tomogram stack (.TIFF):  <https://doi.org/10.17602/M2/M704033>  3D surface file left mandible (.PLY):  https://doi.org/1[0.17602/M2/M704511](https://doi.org/10.17602/M2/M704511)  3D surface file right mandible (.PLY):  https://doi.org/[10.17602/M2/M704508](https://doi.org/10.17602/M2/M704508) |
| “*Moythomasia*” *devonica* BSNS E22113 | Tomogram stack (.TIFF):  <https://doi.org/10.17602/M2/M629178>  3D surface file left mandible (.PLY):  https://doi.org/[10.17602/M2/M671751](https://doi.org/10.17602/M2/M671751) |
| *Osorioichthys marginis* IRSNB P 1340 | Tomogram stack (.TIFF):  [*https://doi.org/*](https://doi.org/)[10.17602/M2/M740679](https://doi.org/10.17602/M2/M740679)  3D surface file right mandible (.PLY):  https://doi.org/[10.17602/M2/M740686](https://doi.org/10.17602/M2/M740686) |
| *Palaeoneiros clackorum* MCZ VPF-5114 | Scan data from Giles et al. (2022):  https://dx.doi.org/10.17602/M2/M420055 |
| “*Kentuckia*” *hlavini* CMNH 9562 | Tomogram stack (.TIFF):  <https://doi.org/10.17602/M2/M568681>  3D surface file left mandible (.PLY):  https://doi.org/[10.17602/M2/M671757](https://doi.org/10.17602/M2/M671757) |
| Actinopterygii n gen n sp CMNH 9560 | Tomogram stack (.TIFF):  <https://doi.org/10.17602/M2/M568382>  3D surface file right mandible (.PLY):  https://doi.org/[10.17602/M2/M671754](https://doi.org/10.17602/M2/M671754) |
| *Tegeolepis clarki* CMNH 8124 | Scan data from Figueroa et al. (2021):  https://doi.org/10.5061/dryad.b8gtht7b7 |
|  |  |
| *Tegeolepis clarki* NHMUK PV P 45312 | Tomogram stack (.TIFF):  <https://doi.org/10.17602/M2/M668651>  3D surface file right mandible (.PLY):  https://doi.org/[10.17602/M2/M669102](https://doi.org/10.17602/M2/M669102) |
| *Limnomis delaneyi* ANSP 23721 | Tomogram stack (.TIFF):  <https://doi.org/10.17602/M2/M669685>  3D surface file right mandible (.PLY):  https://doi.org/1[0.17602/M2/M669691](https://doi.org/10.17602/M2/M669691)  3D surface file left mandible (.PLY):  https://doi.org/[10.17602/M2/M669688](https://doi.org/10.17602/M2/M669688) |
| “*Gonatodus*” *brainerdi* ANSP 6232 | Tomogram stack (.TIFF):  <https://doi.org/10.17602/M2/M683216>  3D surface file left mandible (.PLY):  https://doi.org/[10.17602/M2/M683243](https://doi.org/10.17602/M2/M683243) |
| *Cuneognathus gardineri* NHMD-1235389 | Tomogram stack (.TIFF):  <https://doi.org/10.17602/M2/M671453>  3D surface file right mandible (.PLY):  https://doi.org/[10.17602/M2/M671460](https://doi.org/10.17602/M2/M671460)  3D surface file left mandible (.PLY):  https://doi.org/[10.17602/M2/M671457](https://doi.org/10.17602/M2/M671457) |

**REFERENCES**

Arratia, G. and Cloutier, R. (1996). Reassessment of the morphology of *Cheirolepis canadensis* (Actinopterygii). 165–197. In Schultze, H.-P. and Cloutier, R. (eds). Devonian fishes and plants of Miguasha, Quebec, Canada. Verlag Dr. Frederich Pfeil, Munich, 374 pp.

Arratia, G. (2009). Identifying patterns of diversity of the actinopterygian fulcra. *Acta Zoologica*, *90*, 220-235.

Choo, B., Long, J. A., & Trinajstic, K. (2009). A new genus and species of basal actinopterygian fish from the Upper Devonian Gogo Formation of Western Australia. *Acta Zoologica*, *90*, 194-210.

Choo, B. (2012). Revision of the actinopterygian genus *Mimipiscis* (= *Mimia*) from the Upper Devonian Gogo Formation of Western Australia and the interrelationships of the early Actinopterygii. *Earth and Environmental Science Transactions of the Royal Society of Edinburgh*, *102*(2), 77-104.

Choo, B. (2015). A new species of the Devonian actinopterygian *Moythomasia* from Bergisch Gladbach, Germany, and fresh observations on *M. durgaringa* from the Gogo Formation of Western Australia. *Journal of Vertebrate Paleontology*, *35*(4), e952817.

Clarke, J. M. (1885). On the Higher Devonian fauna of Ontario County, New York. Bulletin of the US Geological Survey, Washington, 3,39–120.

Daeschler, E. B. (2000). An early actinopterygian fish from the Catskill formation (Late Devonian, Famennian) in Pennsylvania, USA. *Proceedings of the Academy of Natural Sciences of Philadelphia*, 181-192.

Dunkle, D. H. (1964). Preliminary description of a paleoniscoid fish from the Upper Devonian of Ohio (Vol. 3, No. 1). Cleveland Museum of Natural History. 3: 1–16.

Dunkle, D. H., & Schaeffer, B. (1973). *Tegeolepis clarki* (Newberry), a palaeonisciform from the Upper Devonian Ohio shale. *Palaeontographica Abteilung A*, 151-158.

Eastman, C. R. (1907). *... Devonic Fishes of the New York Formations* (Vol. 10). New York State Education Department.

Figueroa, R. T., Weinschütz, L. C., & Friedman, M. (2021). The oldest Devonian circumpolar ray-finned fish?. *Biology Letters*, *17*(3), 20200766.

Friedman, M., & Blom, H. (2006). A new actinopterygian from the Famennian of East Greenland and the interrelationships of Devonian ray-finned fishes. *Journal of Paleontology*, *80*(6), 1186-1204.

Gardiner, B. G. (1963). Certain palaeoniscoid fishes and the evolution of the snout in actinopterygians. Bulletin of the British Museum (Natural History), Geology, 8, 254– 325.

Gardiner, B. G. (1984). The relationships of the palaeoniscid fishes, a review based on new specimens of *Mimia* and *Moythomasia* from the Upper Devonian of Western Australia. *Bulletin of the British Museum (Natural History), Geology Series*, *37*(4), 173-428.

Giles, S., Coates, M. I., Garwood, R. J., Brazeau, M. D., Atwood, R., Johanson, Z., & Friedman, M. (2015a). Endoskeletal structure in *Cheirolepis* (Osteichthyes, Actinopterygii), An early ray‐finned fish. *Palaeontology*, *58*(5), 849-870.

Giles, S., Darras, L., Clément, G., Blieck, A., & Friedman, M. (2015b). An exceptionally preserved Late Devonian actinopterygian provides a new model for primitive cranial anatomy in ray-finned fishes. *Proceedings of the Royal Society B: Biological Sciences*, *282*(1816), 20151485.

Giles, S., Feilich, K., Warnock, R. C., Pierce, S. E., & Friedman, M. (2023). A Late Devonian actinopterygian suggests high lineage survivorship across the end-Devonian mass extinction. *Nature Ecology & Evolution*, *7*(1), 10-19.

Gross, W. (1942). Die Fischfaunen des baltischen Devons und ihre biostratigraphische Bedeutung. Korrespondenz-blatt der NaturforscherVereins zu Riga, 64,373–476.

Hussakof, L., & Bryant, W. L. (1918). Catalog of the fossil fishes in the Museum of the Buffalo Society of Natural Sciences. *Buffalo Society of Natural Sciences*, 12, 1-345

Jessen, H. (1968). *Moythomasia nitida* Gross und *M.* cf. *striata* Gross, Devonische palaeonisciden aus dem oberen Plattenkalk der Bergish-Gladbach-Paffrather Mulde (Rheinisches Schiefergebirge). Palaeontographica Abteilung A, 128, 87–114.

Long, J. A. (1988). New palaeoniscoid fishes from the Late Devonian and early Carboniferous of Victoria. Memoir of the Australasian Association of Palaeontologists, 7, 1–64.

Long, J. A., Choo, B., & Young, G. C. (2008). A new basal actinopterygian fish from the Middle Devonian Aztec Siltstone of Antarctica. *Antarctic Science*, 20(4), 393-412.

Lu, J., Giles, S., Friedman, M., den Blaauwen, J. L., & Zhu, M. (2016). The oldest actinopterygian highlights the cryptic early history of the hyperdiverse ray-finned fishes. *Current Biology*, *26*(12), 1602-1608.

Newberry, J. S. (1889). *The Paleozoic fishes of North America* (Vol. 16). US Government Printing Office.

Newman, M. J., Burrow, C. J., den Blaauwen, J. L., & Giles, S. (2021). A new actinopterygian *Cheirolepis* *jonesi* nov. sp. from the Givetian of Spitsbergen, Svalbard. *Norwegian Journal of Geology*, *101*, 1-14.

Pearson, D. M., & Westoll, T. S. (1979). The Devonian Actinopterygian *Cheirolepis* Agassiz. *Earth and Environmental Science Transactions of the Royal Society of Edinburgh*, *70*(13-14), 337-399.

Prokofiev, A. M. (2002). First finding of an articulated actinopterygian skeleton from the Upper Devonian of Siberia and a reappraisal of the family Moythomasiidae Kazantseva, 1971 (Osteichthyes). *Paleontological Research*, *6*(3), 321-327.

Taverne, L. (1997). *Osorioichthys marginis*, “Paléonisciforme” du Famennien de Belgique, et la phylogénie de Actinoptérygiens dévoniens (Pisces). Bulletin de l’Institut Royal des Sciences Naturelles de Belgique 67: 57–78.

Swartz, B. A. (2009). Devonian actinopterygian phylogeny and evolution based on a redescription of *Stegotrachelus finlayi*. *Zoological Journal of the Linnean Society*, *156*(4), 750-784.

Zhu, M., Yu, X., Wang, W., Zhao, W., & Jia, L. (2006). A primitive fish provides key characters bearing on deep osteichthyan phylogeny. *Nature*, *441*(7089), 77-80.

Zhu, M., Wang, W. and Yu, X. (2010). *Meemannia eos*, a basal sarcopterygian fish from the Lower Devonian of China–expanded description and significance. 199–214. In Elliot, D. K., Maisey, J. G., Yu, K. and Miao, D. (eds). Morphology, Phylogeny and Paleobiogeography of Fossil Fishes. Verlag, Dr. Friedrich Pfeil, Munich, 472 pp.

**Supplementary figures**


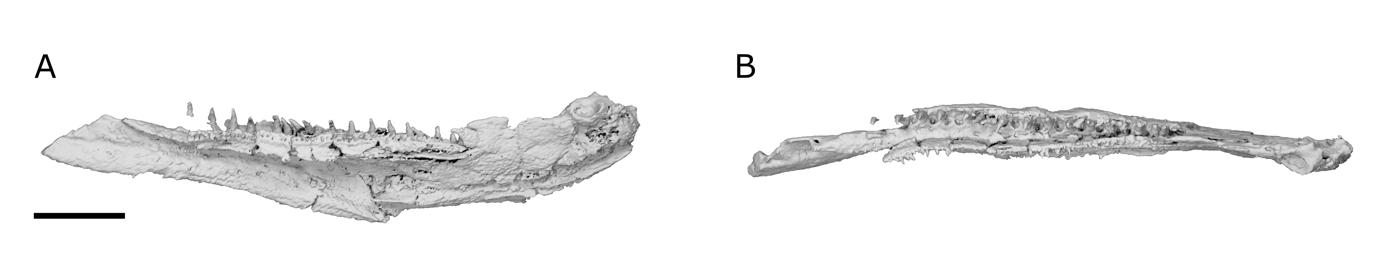


**Supplementary Figure 1. Mandibles of *Cheirolepis trailli* NHMUK PV P 1370 prior to retrodeformation. a,** right mandible in medial view. **b,** right mandible in dorsal view. Scale bar = 5 mm.


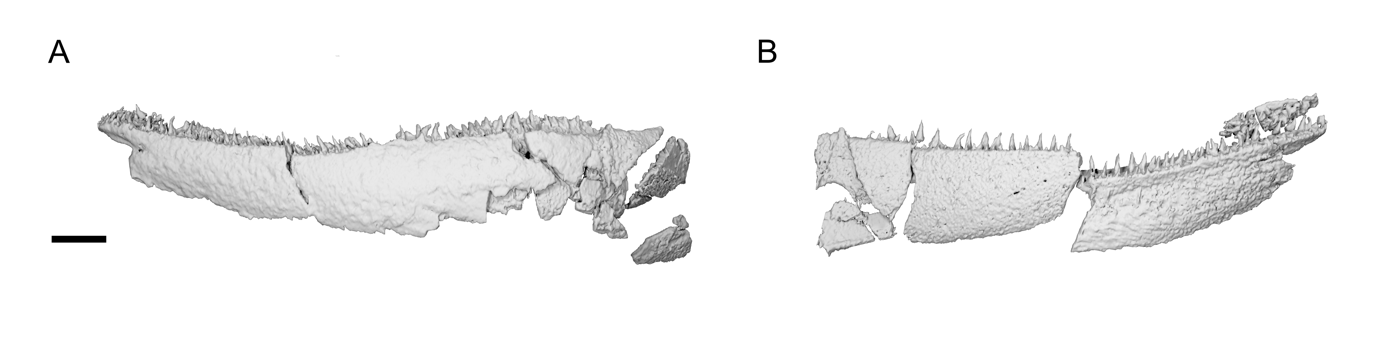


**Supplementary Figure 2. Mandibles of *Cheirolepis jonesi*, PMO 235.121 prior to retrodeformation. a,** left mandible in lateral view. **b,** right mandible in lateral view. Scale bar = 5 mm.

**
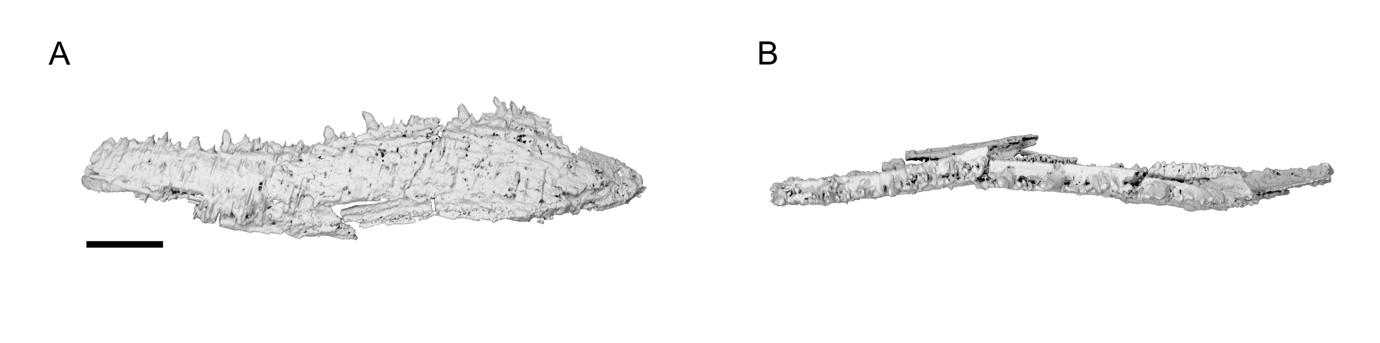
**

**Supplementary Figure 3. Mandibles of *Limnomis delaneyi* ANSP 23721 prior to retrodeformation. a,** left mandible in lateral view. **b,** right mandible in lateral view. Scale bar = 1 mm.
